# Characterization and Expression Analysis of β-Glucosidase Gene Under Abiotic Stresses in Pepper (*Capsicum annuum* L.)

**DOI:** 10.3390/genes16080889

**Published:** 2025-07-27

**Authors:** Jing Wang, Jiaxin Huang, Xu Jia, Zhenxin Hao, Yuancai Yang, Ruxia Tian, Yanping Liang

**Affiliations:** College of Horticulture, Shanxi Agricultural University, Jinzhong 030801, China; wangjing315@sxau.edu.cn (J.W.); 15541217978@163.com (J.H.); xxu_0259@163.com (X.J.); 15649549456@163.com (Z.H.); m19834998834@163.com (Y.Y.); tianruxia@sxau.edu.cn (R.T.)

**Keywords:** pepper, β-glucosidases, bioinformatics analysis, expression analysis

## Abstract

**Background**: Pepper (*Capsicum annuum* L.) is highly susceptible to various abiotic stresses during their growth and development, leading to severe reductions in both yield and quality. β-Glucosidase (BGLU) is widely involved in plant growth and development, as well as in the response to abiotic stress. **Methods**: We performed a genome-wide identification of pepper *BGLU* (*CaBGLU*) genes. Phylogenetic analysis included BGLU proteins from *Arabidopsis*, tomato, and pepper. Gene structures, conserved motifs, and promoter cis-elements were analyzed bioinformatically. Synteny within the pepper genome was assessed. Protein-protein interaction potential was predicted. Gene expression patterns were analyzed across tissues and under abiotic stresses using transcriptomic data and qRT-PCR. Subcellular localization of a key candidate protein CaBGLU21 was confirmed experimentally. **Results**: We identified 32 *CaBGLU* genes unevenly distributed across eight chromosomes. Phylogenetic classification of 99 BGLU proteins into 12 subfamilies revealed an uneven distribution of CaBGLUs across six subfamilies. Proteins within subfamilies shared conserved motifs and gene structures. *CaBGLU* promoters harbored abundant light-, hormone- (MeJA, ABA, SA, GA), and stress-responsive elements (including low temperature). A duplicated gene pair (*CaBGLU19*/*CaBGLU24*) was identified. 27 CaBGLU proteins showed potential for interactions. Expression analysis indicated *CaBGLU5* and *CaBGLU30* were mesophyll-specific, while *CaBGLU21* was constitutively high in non-leaf tissues. *CaBGLU21* was consistently upregulated by cold, heat, and ABA. Subcellular localization confirmed CaBGLU21 resides in the tonoplast. **Conclusions**: This comprehensive analysis characterizes the pepper *BGLU* gene family. *CaBGLU21*, exhibiting constitutive expression in non-leaf tissues, strong upregulation under multiple stresses, and tonoplast localization, emerges as a prime candidate gene for further investigation into abiotic stress tolerance mechanisms in pepper. The findings provide a foundation for future functional studies and stress-resistant pepper breeding.

## 1. Introduction

Pepper (*Capsicum annuum* L.), a member of the Solanaceae family, is a widely cultivated annual or short-lived perennial plant globally. Its fruits are used not only as fresh vegetables and spices but also serve as raw materials for the pharmaceutical and cosmetic industries [1]. However, pepper production is frequently confronted with various abiotic stresses, such as drought, soil salinization, and high temperatures, which significantly reduce both yield and quality. Therefore, identifying stress-resistant genes in pepper and investigating its stress tolerance mechanisms are crucial.

β-Glucosidase (BGLU), also known as β-D-glucoside glucohydrolase, was first discovered in bitter almonds [2,3]. In higher plants, BGLU genes exist as multigene families and extensively participate in defense responses [4], cell wall lignification [5,6], and key physiological processes including plant hormone signaling activation and secondary metabolism [7,8,9]. The glycoside hydrolase (GH) family comprises 166 subfamilies, classified into GH1, GH3, GH5, and GH116 [10]. Among these, GH1 represents the largest subfamily, with the majority of identified plant BGLUs belonging to this group [11]. To date, BGLU gene families have been systematically characterized in several plant species, including *Arabidopsis thaliana* (47 members) [12], *Oryza sativa* (40) [13], *Medicago truncatula* (51) [14], *Zea mays* (26) [15], and *Brassica rapa* (64) [16]. Phylogenetic analysis revealed that *Arabidopsis* AtBGLU45, AtBGLU46, and AtBGLU47 cluster with the coniferin β-glucosidase gene from *Pinus contorta*, suggesting their potential involvement in lignification through monolignol glucoside hydrolysis [17]. Functional studies demonstrate that suppression of *OsBGLU6* in rice leads to multiple phenotypic consequences: dwarfism, reduced leaf ABA content, enhanced drought sensitivity, decreased photosynthetic rate, and elevated intercellular CO_2_ concentration, collectively impairing drought tolerance [18]. *SrBGLU16* is substantially upregulated in response to dark treatment [19]. Beyond their established roles in cell wall metabolism and phytohormone activation, members of the BGLU gene family are crucial players in plant stress defense responses.

The BGLU gene family in pepper has not yet been systematically characterized, significantly hindering the functional investigation of its members. This study performed genome-wide identification of the pepper BGLU gene family using its complete genome sequence and comparative genomic data from the characterized BGLU families of *A*. *thaliana* and *Solanum lycopersicum*. We comprehensively characterized the identified BGLU members through analyses of their physicochemical properties, gene structures, phylogenetic relationships, chromosomal distributions, cis-acting elements, and syntenic relationships. Expression patterns of pepper BGLU genes in various tissues and under stress treatments were analyzed using qRT-PCR. Subcellular localization of CaBGLU21 was also determined. This study provides valuable insights into the roles of the pepper BGLU gene family in growth, development, and stress responses, establishing a foundation for breeding stress-resistant pepper cultivars.

## 2. Materials and Methods

### 2.1. Materials

Seeds of the heat-tolerant pepper cultivar ‘17CL30’ were surface-sterilized with 55 °C hot water for 15 min, germinated at 28 °C until radicle emergence, and subsequently grown in climate-controlled growth chambers under a 16 h light/8 h dark photoperiod with day/night temperatures of 28 °C and 24 °C, respectively.

At the six-true-leaf stage, pepper plants were subjected to various treatments including low temperature (10 °C), high temperature (42 °C), ABA (30 μM), IAA (2 μM), GA (2 μM), H_2_O_2_ (30 mM), NaCl (200 mM), and JA (10 μM), with plants under normal growth conditions serving as the control. For temperature treatments, whole plants were transferred to climate-controlled chambers. For chemical treatments (ABA, IAA, GA, H_2_O_2_, NaCl, JA), plants were removed from soil, their roots were gently washed with deionized water, and then carefully transferred to Hoagland nutrient solution for 24 h acclimation. After adaptation, the chemical agents were directly supplemented into the existing nutrient solution at specified concentrations. The concentrations of the tested agents and time windows were determined based on Pepper Hub database protocols (http://pepperhub.hzau.edu.cn/, accessed on 20 March 2024). Leaf samples were collected from each treatment at 1, 1.5, 3, 6, 12, and 24 h post-treatment, immediately frozen in liquid nitrogen, and stored at −80 °C. Three biological replicates were included per treatment.

Roots, stems, leaves (at the six-leaf one-heart stage), flowers (adult plant stage), and fruits (green mature stage) of pepper were collected, flash-frozen in liquid nitrogen, and stored at −80 °C for tissue-specific expression analysis.

### 2.2. Methods

#### 2.2.1. The Identification of the *CaBGLU* Gene Family

To identify potential members of the BGLU family in pepper, protein sequences of the Zunla-1 cultivar were retrieved from the Pepper Genome Platform (https://plantgarden.jp/en/list/t4072/genome/t4072.G002, accessed on 1 November 2024). Concurrently, BGLU protein sequences from tomato (*S*. *lycopersicum*) and *A*. *thaliana* were obtained from the NCBI database (https://www.ncbi.nlm.nih.gov/, accessed on 1 November 2024) to construct a local protein database. Three complementary approaches were employed to identify pepper *BGLU* genes. First, the β-glucosidase domain Hidden Markov Model (HMM) profile (PF00232) was acquired from the Pfam database (http://pfam.xfam.org/, accessed on 1 November 2024). Second, a custom HMM was built using hmmbuild (Ubuntu environment), followed by screening the Zunla-1 proteome with HMMER’s hmmsearch program (v.3.2.1) to identify putative BGLU proteins (E < 1 × 10^−5^). Third, BLASTP analysis was performed using BioEdit (v7.0.5.3), aligning *Arabidopsis* and tomato BGLU sequences against the pepper proteome to detect homologous genes. Candidate proteins from all three methods were consolidated, and redundancies were removed. Finally, conserved domains were validated using SMART (http://smart.embl-heidelberg.de/, accessed on 2 November 2024) and CDD (https://www.ncbi.nlm.nih.gov/cdd/, accessed on 2 November 2024). Only sequences containing the Glyco_hydro_1 domain were retained as bona fide members of the BGLU family in pepper.

#### 2.2.2. Physicochemical Characterization and Subcellular Localization Prediction of the CaBGLU Gene Family

The physicochemical properties of pepper BGLU family members—including amino acid count, molecular weight, isoelectric point (pI), instability index, aliphatic index, and grand average of hydropathicity (GRAVY)—were analyzed using ExPASy (https://www.expasy.org/, accessed on 4 November 2024). Subcellular localization was predicted using Cell-PLoc 2.0 (http://www.csbio.sjtu.edu.cn/bioinf/Cell-PLoc-2/, accessed on 4 November 2024).

#### 2.2.3. Chromosomal Localization and Phylogenetic Analysis of the CaBGLU Gene Family

Chromosomal localization was analyzed using the MG2C online tool (http://mg2c.iask.in/mg2c_v2.0/, accessed on 6 November 2024). To investigate evolutionary relationships among *BGLU* genes, protein sequences from *A*. *thaliana*, *S*. *lycopersicum*, and *C*. *annuum* were aligned using Clustal X (v1.83). A maximum likelihood (ML) phylogenetic tree was then constructed with MEGA-X (v10.2.6) using default parameters. The resulting tree was visualized and annotated using iTOL (https://itol.embl.de/, accessed on 6 November 2024).

#### 2.2.4. Prediction of Conserved Motifs, Gene Structures, and Cis-Acting Elements in the CaBGLU Gene Family

Conserved motif analysis of pepper BGLU proteins was performed using MEME Suite (http://meme-suite.org/tools/meme, accessed on 7 November 2024) with 10 output motifs and default parameters.

Promoter regions (2000 bp upstream of transcription start sites) were extracted from pepper BGLU genes using TBtools (v2.210). Cis-regulatory elements were predicted using the PlantCARE database (http://bioinformatics.psb.ugent.be/webtools/plantcare/html/, accessed on 7 November 2024), followed by visualization with TBtools (v2.210).

#### 2.2.5. Collinearity Analysis of CaBGLU Gene Family

Genome-wide synteny analysis of *BGLU* family members was performed using TBtools (v2.210), employing both intraspecific (within *C*. *annuum*) and interspecific (across related species) comparisons through the “One Step MCScanX” and “Advanced Circos” plugins.

#### 2.2.6. Protein–Protein Interaction Network Analysis of the CaBGLU Gene Family

Protein–protein interaction networks of the CaBGLU family were predicted using the STRING database (https://string-db.org/, accessed on 8 November 2024).

#### 2.2.7. Expression Profile Analysis of *CaBGLU* Gene Family Members

Expression profiles of *C*. *annuum* across leaf, flower, and fruit developmental stages, plus abiotic stress and phytohormone treatments, were retrieved from PepperHub (http://pepperhub.hzau.edu.cn/, accessed on 20 November 2024). Data were preprocessed (normalization, filtering) using Microsoft Excel (v16.85) and visualized as hierarchical clustered heatmaps through TBtools (v2.210), distinguishing tissue-specific and stress-responsive expression patterns.

#### 2.2.8. qRT-PCR Analysis

Total RNA was extracted from *C*. *annuum* leaves using the Polysaccharide-Polyphenol Plant RNA Kit (Tiangen Biotech, Beijing, China). First-strand cDNA was synthesized from 1 μg total RNA using the UnionScript First Strand cDNA Synthesis Mix for qPCR (with dsDNase; Genesand Biotech, Beijing, China) and stored at −20 °C. Gene-specific primers (Table S1) were designed with Primer Premier 6.0, with *β-Actin* as the reference gene. Quantitative PCR was performed in 20 μL reactions containing 10 μL AceQ qPCR SYBR Green Master Mix (Vazyme, Nanjing, China), 0.4 μM of each primer, and 2 μL cDNA template. Thermal cycling conditions on a QuantStudio 5 system (Applied Biosystems) (Thermo Fisher Scientific, Waltham, MA, USA) were 95 °C for 30 s; 40 cycles of 95 °C for 10 s; and 60 °C for 30 s. Three biological replicates, each with technical triplicates, were analyzed per sample. Relative expression was calculated using the 2^−ΔΔCT^ method. Statistical significance (one-way ANOVA, *p* < 0.05) was determined using SPSS Statistics v27.0 (IBM), with data visualized in Origin 2021.

#### 2.2.9. Subcellular Localization of CaBGLU21

The subcellular localization vector for CaBGLU21 was constructed as follows. The target gene was amplified from cDNA of heat-stressed (42 °C, 24 h) *C*. *annuum* seedling leaves using primers pART-CAM-**e**GFP-*CaBGLU21*-F/R. The purified PCR product was cloned into the pART-CAM-eGFP expression vector via homologous recombination using *Xho*I/*Eco*RI restriction sites. The recombinant plasmid pART-CAM-eGFP-*CaBGLU21* was transformed into *Escherichia coli* DH5α competent cells. Positive clones were selected on LB/kanamycin plates (37 °C, 16 h), verified by colony PCR, and sequenced. For transient expression, both recombinant and empty vectors were electroporated into *Agrobacterium tumefaciens* GV3101. Bacterial suspensions were infiltrated into the abaxial surfaces of fully expanded leaves from 40-day-old *Nicotiana benthamiana* plants. After overnight incubation at 22 °C in darkness, plants were maintained under normal growth conditions for 2–3 days. Fluorescence signals were ultimately visualized using a confocal laser scanning microscope (Leica TCS SP8; Wetzlar, Germany). GFP was excited at 488 nm and emission collected at 500–550 nm; mCherry was excited at 561 nm and emission collected at 570–620 nm. Chloroplast autofluorescence was detected at 640 nm under 675 nm excitation.

## 3. Results

### 3.1. Identification and Physicochemical Characterization of the CaBGLU Gene Family

This study systematically identified 32 *BGLU* family genes in pepper, which were designated as CaBGLU1 through CaBGLU32 according to their chromosomal locations. Comprehensive physicochemical characterization revealed considerable diversity in the molecular properties of these CaBGLU proteins (Table S2). Protein lengths vary from 127 to 631 amino acids, corresponding to molecular weights ranging between 14.65 and 71.72 kDa. pI points span from 5.09 to 9.64, classifying the proteins into 17 acidic (pI < 7) and 15 alkaline (pI > 7) groups. Instability index analysis showed that 29 members are stable proteins (index < 40), while CaBGLU25, CaBGLU26, and CaBGLU29 are predicted to be unstable (index > 40). The aliphatic indices (67.83–101.54) suggest good thermostability, and the GRAVY values for all members except CaBGLU29 indicate predominant hydrophilicity. Subcellular localization predictions indicated that 27 members (84.4% of the total) were exclusively localized to the vacuole, while 5 members (CaBGLU11, CaBGLU14, CaBGLU15, CaBGLU20, and CaBGLU29) were predicted to localize to both the chloroplast and vacuole, though the predictions did not specify their exact suborganellar positions, implicating their potential roles in organelle-specific metabolic processes.

### 3.2. Chromosomal Localization and Phylogenetic Analysis

**Figure 1:**
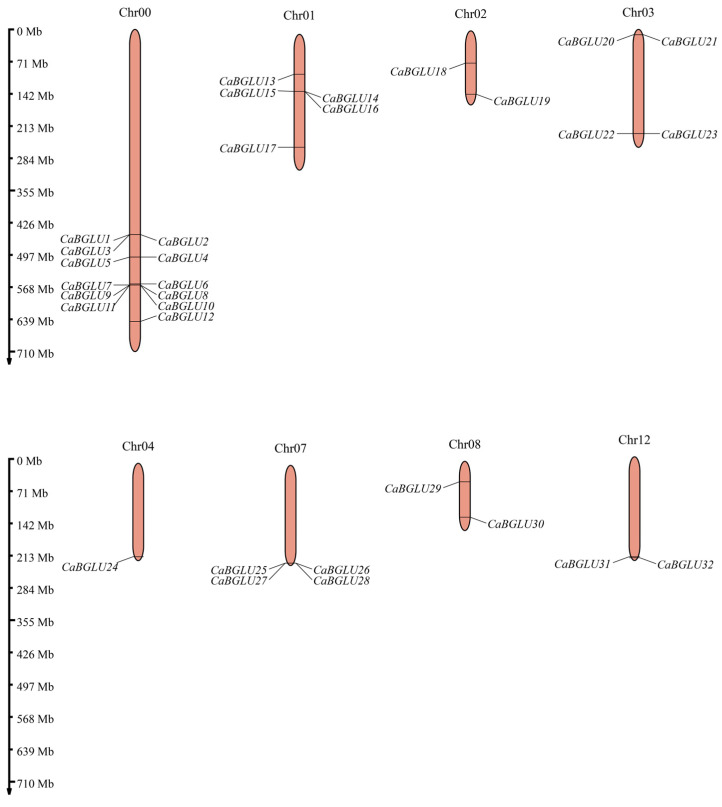
Distribution of the *CaBGLU* gene family on 8 chromosomes in pepper.

The 32 *CaBGLU* genes were unevenly distributed across eight chromosomes of *C*. *annuum* (Figure 1), with significant clustering observed. Chromosome 0 contained the highest density (12 genes), followed by chromosome 1 (5 genes). Chromosomes 3 and 7 each harbored four genes, while chromosomes 2, 8, and 12 contained two genes each. Chromosome 4 possessed only a single *CaBGLU* member. This distribution pattern suggests chromosomal hotspots for gene duplication events, particularly on chromosome 0, potentially driving the expansion of this gene family in pepper.

**Figure 2:**
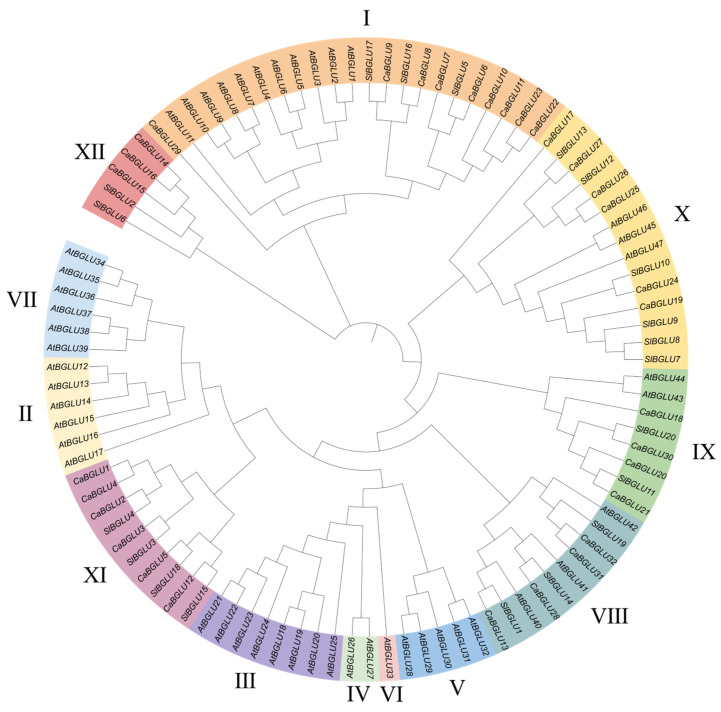
Phylogenetic tree of BGLU family members of *A*. *thaliana* (*At*), *S*. *lycopersicum* (*Sl*), and *C*. *annuum* (*Ca*).

To investigate the evolutionary relationships of the pepper BGLU family, we constructed a phylogenetic tree comprising BGLU proteins from three species: *A*. *thaliana* (47 members), *S*. *lycopersicum* (20 members), and *C*. *annuum* (32 members) (Figure 2). Based on the clustering patterns observed in the *Arabidopsis* and tomato BGLU families, the 99 BGLU proteins were classified into twelve subfamilies (I–XII). The analysis revealed that CaBGLU proteins are unevenly distributed across six subfamilies: Subfamily I contains the largest number of CaBGLUs (9 members); Subfamilies VIII and IX each include 4 CaBGLU proteins; Subfamilies X and XI each contain 6 CaBGLU proteins; and Subfamily XII comprises 3 CaBGLU members. Notably, Subfamilies II–VII lack any CaBGLU representatives.

### 3.3. Analysis of Conserved Motifs and Exon-Intron Structures

**Figure 3:**
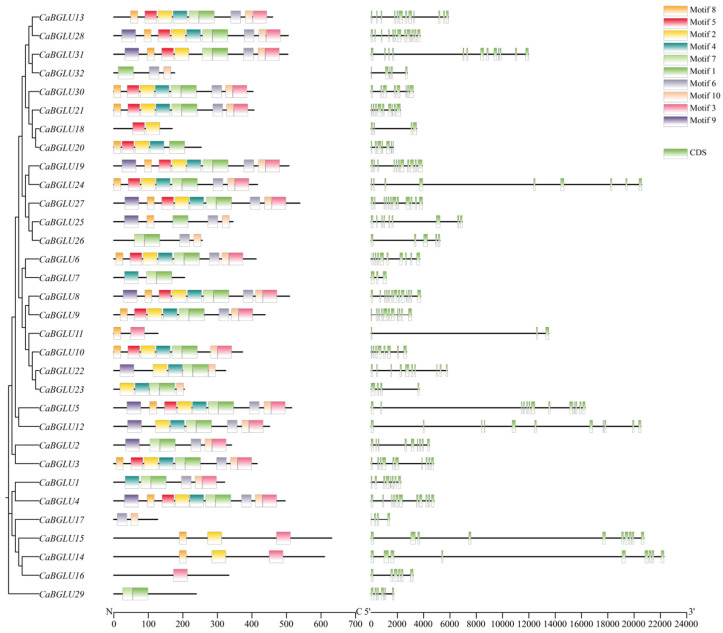
Prediction of BGLU family protein structure in pepper.

Analysis of conserved motif distribution among BGLU proteins revealed that each member contained 1 to 10 motifs, with differential conservation across the family (Figure 3). Motif 1 was the most prevalent, present in 26 CaBGLU proteins (81.3% of family members), followed by Motif 10 (24 proteins; 75%). Motifs 2, 3, 6, and 7 exhibited intermediate conservation (21–23 proteins; 65.6–71.9%), whereas Motifs 4, 5, and 8 occurred in 17–20 members (53.1–62.5%). Notably, Motif 9 demonstrated the lowest frequency, appearing in only 11 CaBGLU proteins (34.4%). Six proteins—CaBGLU4, CaBGLU5, CaBGLU8, CaBGLU19, CaBGLU27, and CaBGLU28—contained the complete set of 10 motifs, suggesting they may represent ancestral forms with comprehensive functional domains. In stark contrast, CaBGLU16 possessed only a single motif (Motif 1), indicating either extreme functional specialization or potential degradation of ancestral domains during evolution.

Genomic annotation of the *C*. *annuum* genome revealed substantial variation in exon-intron architecture among *CaBGLU* family members (Figure 3). The exon number ranged from 3 to 13. *CaBGLU5*, *CaBGLU8*, *CaBGLU28*, and *CaBGLU31* exhibited the highest number of exons (13 each), indicating complex structural organization. Conversely, *CaBGLU7* and *CaBGLU11* possessed the simplest structures, with only 3 exons, suggesting potential evolutionary streamlining or alternative splicing variants.

### 3.4. Analysis of Cis-Acting Elements in the Promoter Region

**Figure 4:**
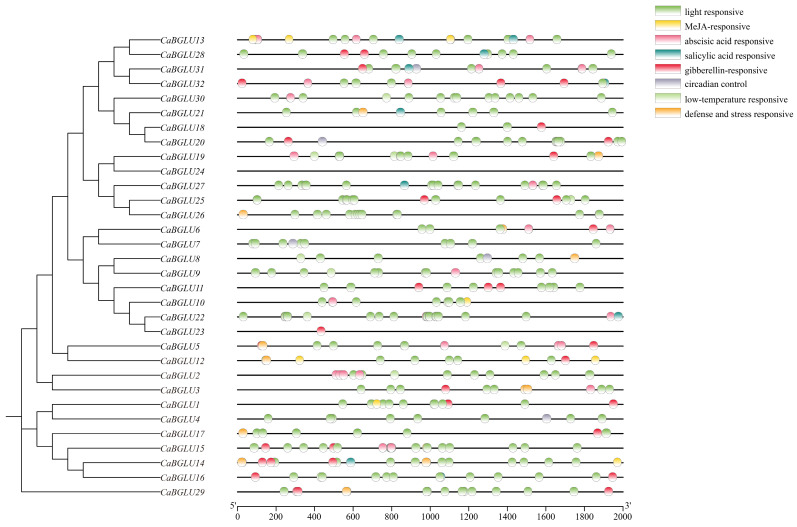
Cis-acting elements of the pepper BGLU family.

Analysis of cis-acting elements within the 2000 bp promoter regions upstream of *CaBGLU* genes identified eight major functional categories: light-responsive, MeJA-responsive, abscisic acid (ABA)-responsive, salicylic acid (SA)-responsive, gibberellin (GA)- responsive, circadian control, low-temperature responsive, and defense/stress-responsive elements (Figure 4). Notably, light-responsive elements were the most abundant, present in all *CaBGLU* promoters with the exception of *CaBGLU24*, suggesting photoregulation as a central regulatory mechanism for this gene family. The co-enrichment of stress- and hormone-related elements—particularly MeJA-, ABA-, and defense-responsive motifs—indicates that pepper *BGLU* genes participate in plant–pathogen interactions and adaptation to environmental stresses, including low temperature and biotic stressors.

### 3.5. Collinearity Analysis of the CaBGLU Gene Family

**Figure 5:**
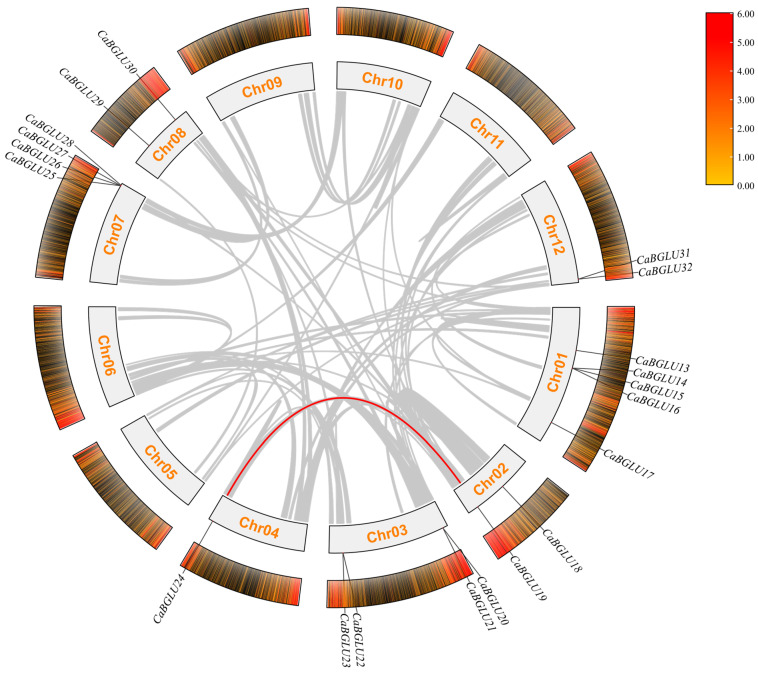
The inter-genomic collinearity of *CaBGLU* genes in pepper. *Circos plot* structure: Innermost ring (Chr00–12) represents chromosomes; yellow band indicates gene density; outermost ring shows chromosomal positions of 32 *CaBGLU* genes. *Collinearity relationships*: Gray lines denote genome-wide duplication events; red lines indicate *CaBGLU*-specific duplication events.

**Figure 6:**
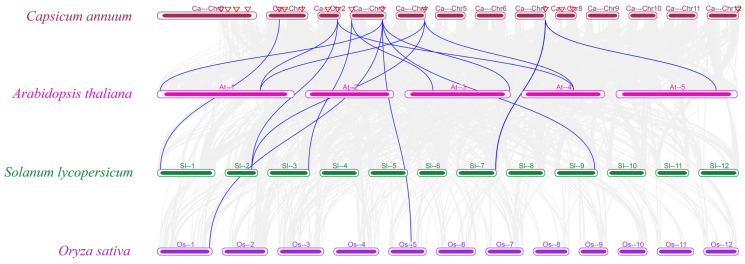
Synteny analyses of the *BGLU* genes between *C*. *annuum* and three representative plants (*A*. *thaliana*, *S*. *lycopersicum,* and *O*. *sativa*). Gray background lines indicate synteny blocks across all genomes. Blue lines highlight syntenic *C. annuum* BGLU gene pairs with *A*. *thaliana*, *S*. *lycopersicum*, and *O*. *sativa*.

Intraspecific collinearity analysis of pepper *CaBGLU* genes revealed a duplication event between *CaBGLU19* (chromosome 2) and *CaBGLU24* (chromosome 4) (Figure 5). Interspecies collinearity analysis of BGLU genes in *C*. *annuum*, *A*. *thaliana*, *S*. *lycopersicum*, and *O*. *sativa* demonstrated extensive genomic homology (Figure 6)*. CaBGLU* genes showed the highest number of homologous pairs with *A. thaliana* (eight pairs), followed by *S. lycopersicum* (6 pairs), and the lowest with *O. sativa* (two pairs).

### 3.6. Protein–Protein Interaction Network Analysis of the CaBGLU Family

**Figure 7:**
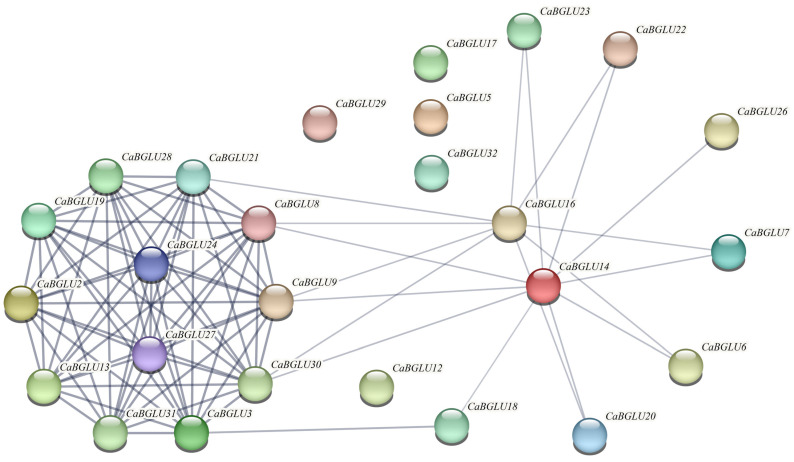
Predicted protein–protein interaction network of the BGLU gene family. Nodes represent gene products, with color intensity indicating interaction degree (darker = higher connectivity). Line thickness corresponds to interaction strength (thicker = stronger associations). Edge saturation scales with confidence scores.

Protein–protein interaction network analysis of CaBGLU family members revealed that CaBGLU5, CaBGLU12, CaBGLU17, CaBGLU29, and CaBGLU32 showed no detectable interactions, whereas CaBGLU8, CaBGLU9, and CaBGLU30 exhibited the highest connectivity (13) (Figure 7), suggesting functional divergence within the family.

### 3.7. Expression Profiling of CaBGLU Genes Across Pepper Tissues and Stress Treatments

**Figure 8:**
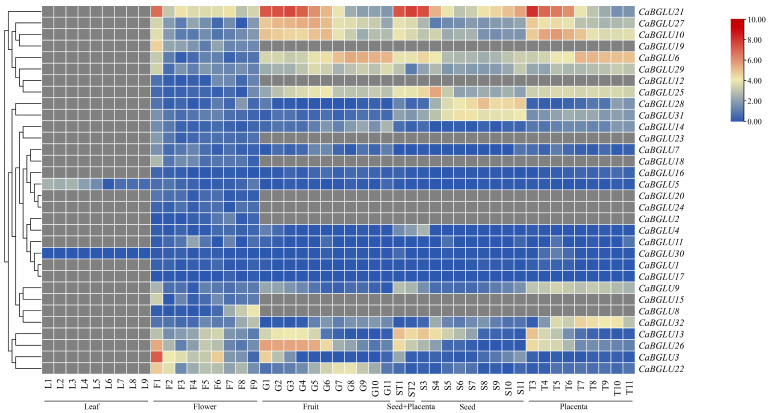
Expression analysis of *CaBGLU* in different tissues in pepper.

Transcriptomic analysis of 32 *CaBGLU* genes across five pepper tissues—leaves, flowers, fruits, seeds, and placenta—revealed distinct expression patterns (Figure 8). Only *CaBGLU5* and *CaBGLU30* were detected in leaves, with potential involvement in leaf-specific processes such as photosynthetic metabolism or defense-related activities in mesophyll cells. *CaBGLU21* exhibited constitutively high expression in all non-leaf tissues, indicating potential housekeeping roles. *CaBGLU6*, *CaBGLU10*, and *CaBGLU27* showed elevated expression in fruits and placenta, potentially associated with defense compound biosynthesis A. *CaBGLU2*, *CaBGLU8*, *CaBGLU15*, *CaBGLU18*, *CaBGLU19*, *CaBGLU20*, *CaBGLU23*, and *CaBGLU24* displayed flower-specific expression, supporting roles in reproductive development. *CaBGLU26* was predominantly expressed in fruits, suggesting the regulation of ripening processes.

**Figure 9:**
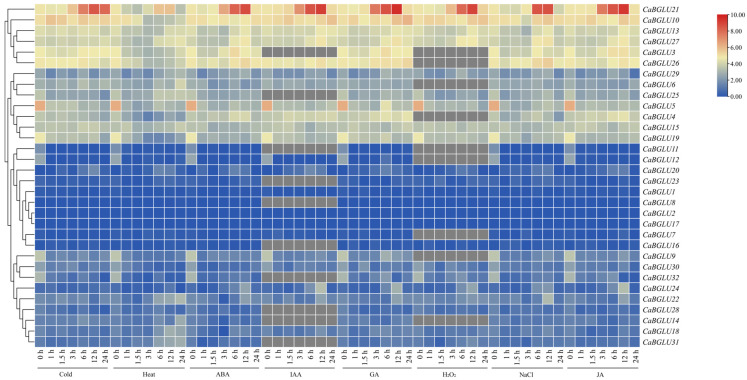
Expression analysis of *CaBGLU* in different treatments in pepper.

Transcriptomic analysis of *CaBGLU* gene expression under multiple abiotic stresses—cold, heat, ABA, IAA, GA, H_2_O_2_, NaCl, and JA—revealed distinct regulatory patterns (Figure 9). *CaBGLU21* was upregulated under all treatments, peaking at 12 h post-induction, suggesting function as a possible stress adaptation regulator. *CaBGLU3*, *CaBGLU4*, *CaBGLU5*, *CaBGLU6*, *CaBGLU10*, *CaBGLU13*, *CaBGLU15*, *CaBGLU19*, *CaBGLU25*, *CaBGLU26*, *CaBGLU27*, and *CaBGLU29* exhibited sustained high expression across most stresses, indicating involvement in broad-spectrum stress adaptation. From 12 to 24 h post-treatment, the expression levels of most *CaBGLU* genes (e.g., *CaBGLU3*, *CaBGLU4*, *CaBGLU21*) exhibited a significant decline under all stress conditions (Figure 9). Notably, *CaBGLU21*, despite its initial upregulation, showed a >50% reduction in transcript abundance by 24 h in response to cold, heat, and ABA treatments. The remaining *CaBGLU* genes showed minimal transcriptional changes, potentially maintaining basal physiological functions.

### 3.8. Relative Expression Levels of CaBGLU Genes in Pepper

**Figure 10:**
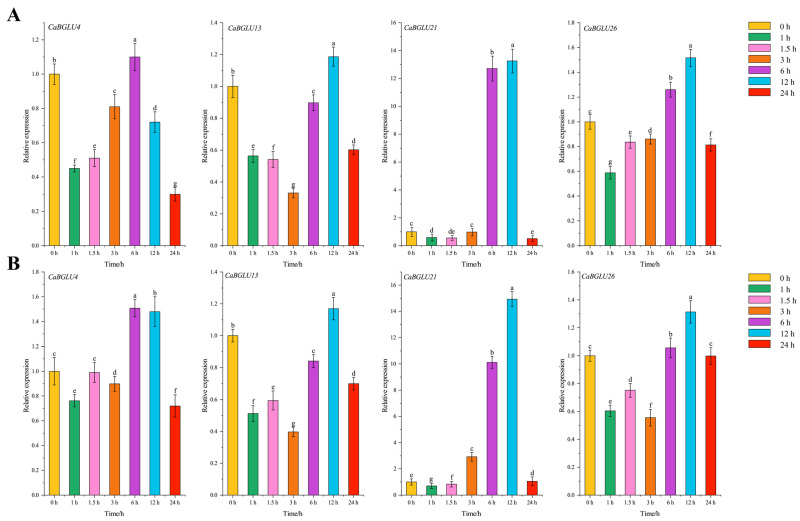
Relative expression of four representative CaBGLU genes under NaCl and ABA treatments. (**A**) Expression patterns under NaCl treatment. (**B**) Expression patterns under ABA treatment. Data are presented as mean ± SD (*n* = 3). Different letters indicate significant differences (*p* < 0.05) according to one-way ANOVA.

To investigate the expression profiles of *CaBGLU* genes, four randomly selected members (*CaBGLU4*, *CaBGLU13*, *CaBGLU21,* and *CaBGLU26*) were analyzed via qRT-PCR under NaCl and ABA treatments. The results demonstrated a biphasic response in relative expression levels (Figure 10). The expression dynamics of *CaBGLU* genes revealed a biphasic response. Statistical analysis revealed no significant differential expression (*p* > 0.05) for these genes during the early stress response phase (1–3 h post-treatment). However, significant upregulation (*p* < 0.05) was detected at 6 h for *CaBGLU4* and at 12 h for *CaBGLU13*, *CaBGLU21*, and *CaBGLU26* (Figure 10). This suggests that the selected *CaBGLU* genes might function in the middle-to-late phases of stress response, thus potentially being involved in adaptation processes.

### 3.9. Subcellular Localization of CaBGLU21

**Figure 11:**
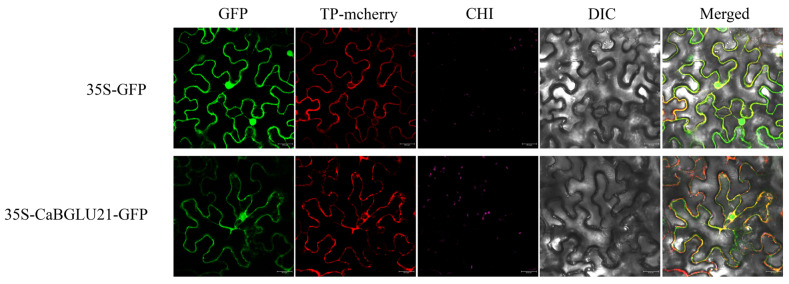
Subcellular localization analysis of CaBGLU21 in tobacco leaves. Panels show (left to right): GFP fluorescence, TP-mCherry, chloroplast autofluorescence, bright-field, and merged channels. Scale bar = 25 μm.

To validate subcellular localization predictions, the *CaBGLU21*-eGFP fusion construct (pART-CAM-eGFP vector) and empty eGFP control were transiently expressed in tobacco epidermal cells. Tonoplast marker mCherry (TP-mCherry) confirmed organelle positions. Confocal microscopy revealed tonoplast localization of CaBGLU21 (Figure 11).

## 4. Discussion

Pepper, as a widely cultivated vegetable crop in China, is vulnerable to both biotic and abiotic stresses that limit yield and quality, and faces escalating threats from extreme heat events under climate change. While β-glucosidases (BGLUs) are known to regulate plant physiological processes—particularly in secondary metabolism and stress responses—and have been extensively studied in *Arabidopsis*, tomato, rice, and maize, they remain uncharacterized in pepper. In this study, we systematically identified pepper *BGLU* genes, analyzed their phylogenetic relationships, promoter cis-elements, and collinearity patterns, and compared their expression profiles across different tissues, stress treatments, and hormone treatments. Additionally, we conducted subcellular localization assays.

Bioinformatics analysis identified 32 *BGLU* family genes in pepper, all predicted to localize to vacuoles or chloroplasts, consistent with findings in *Dendrobium catenatum* [20] and *Z*. *mays* [21]. Given the vacuole’s critical roles in nutrient storage, stress response, and metabolic turnover, coupled with prior evidence that the *Arabidopsis* vacuolar-localized BGLU10 enhances drought tolerance [22], we hypothesize that these genes significantly influence pepper development and stress adaptation. These 32 *CaBGLU* genes are unevenly distributed across eight chromosomes, with most located near telomeres, and encode proteins containing 10 conserved motifs. Most genes were identified in homologous pairs, indicating potential functional redundancy. Phylogenetic analysis grouped them unevenly into six subfamilies, each containing orthologs from tomato, supporting Solanaceae-specific evolutionary conservation.

Cis-acting element prediction revealed that *BGLU* gene promoters are enriched with light-responsive, exogenous ABA- and GA-associated regulatory motifs, suggesting their potential roles in photosynthesis, photomorphogenesis, and hormone signaling. Collinearity analysis further identified 16 syntenic gene pairs between pepper *CaBGLUs* and their orthologs in *A*. *thaliana* and *S*. *lycopersicum*, reflecting evolutionary conservation across these species and indicating potential functional similarity in associated biological processes.

Expression of *BGLU* genes exhibits pronounced tissue specificity, as demonstrated in the *AtBGLU* and *OsBGLU* families where specific members (e.g., *OsBGLU10*, *OsBGLU24*, and *OsBGLU33*) play specialized roles in processes such as seed germination, root elongation, and drought tolerance [23]. Similarly, we observed distinct expression patterns of *CaBGLU* genes across various pepper tissues, indicating their functional diversification in plant growth and development. *CaBGLU5* and *CaBGLU30* exhibited leaf-specific expression, but their precise biological roles require further investigation. Given the functional diversity of leaf cell types—including photosynthesis in mesophyll, stomatal regulation in guard cells, and auxin biosynthesis in vascular tissues—future studies should determine whether these genes localize to specific cell types and participate in defined physiological processes such as carbon metabolism or stress-induced defense responses.

The evolutionary significance of β-glucosidases in plant stress adaptation is underscored by their conservation across diverse plant species. The expansion of the *BGLU* gene family through gene duplication events, such as the *CaBGLU19/CaBGLU24* pair identified in this study, has likely contributed to functional diversification, allowing plants to fine-tune their responses to various environmental stresses. Notably, the retention of stress-responsive cis-elements in *CaBGLU* promoters and the up-regulation of specific members (e.g., *CaBGLU21*) under multiple stresses suggest evolutionary selection for stress adaptation. This functional conservation highlights the fundamental role of BGLU enzymes in hydrolyzing stress-related compounds to activate defense responses, a mechanism that has been evolutionarily optimized to enhance survival in fluctuating environments. *BGLU* genes play crucial roles in plant responses to abiotic stress and hormone signaling [24]. Evidence demonstrates that overexpression of specific *Arabidopsis* genes, such as *AtBGLU10* [22] and *AtBGLU18* [25], enhances tolerance to abiotic stresses like drought and salinity, concomitant with increased ABA levels. Mechanistically, *AtBGLU18* [25] and *AtBGLU33* [26] hydrolyze glucose-conjugated ABA, thereby elevating free ABA levels and activating downstream ABA responses, including drought tolerance. Similarly, ABA treatment significantly upregulates specific *MsBGLUs* (*MsBGLU99*, *MsBGLU63*, *MsBGLU62*, *MsBGLU78*, *MsBGLU49*) in *Medicago sativa* [27]. In maize, *ZmBGLU1* enhances salt stress tolerance, potentially via defense responses or phytohormone activation [15]. *FtBGLU34* and *FtBGLU38* in *Tartary buckwheat* are markedly stress-induced, with *FtBGLU29* exhibiting the strongest upregulation under ABA treatment. The decline in *CaBGLU* transcript levels from 12 to 24 h post-induction may reflect a feedback regulation mechanism to prevent prolonged metabolic costs under stress. Similar transient expression patterns have been reported for *AtBGLU18* in *Arabidopsis* under ABA treatment, where rapid mRNA turnover limits sustained hormone activation [25].

The BGLU gene family enhances plant stress resilience primarily by activating phytohormone crosstalk, while also modulating cell wall remodeling and secondary metabolism [28]. Further supporting this conserved role, *MtBGLU21*, *MtBGLU22*, *MtBGLU28*, and *MtBGLU30* in *M*. *truncatula* are strongly induced by both abiotic stress and hormonal treatments [14]. Consistent with these findings in other species, our study identified that *CaBGLU4/13/21/26* reached peak expression at 6–12 h post-treatment, indicating roles in sustained stress adaptation rather than immediate signaling. Early response genes (1–3 h) may require further investigation.

While this study provides insights into *CaBGLU* stress responses, certain technical limitations warrant acknowledgment. The initial protocol involving root washing and transfer may introduce transient stress artifacts. Although our optimized hydroponic acclimation procedure minimized this effect, future studies should employ non-invasive agent application methods. Additionally, while agent concentrations followed database protocols, dose response assays including IC50 determination could further refine treatment precision.

## 5. Conclusions

In this study, we systematically identified 32 *CaBGLU* genes within the pepper (*C*. *annuum*) genome. Comprehensive analyses were conducted to characterize these genes, including their physicochemical properties, genomic organization (chromosomal distribution, gene structure, collinearity), evolutionary relationships (phylogenetics), regulatory potential (cis-acting elements), protein interaction networks, and expression dynamics (tissue-specificity and responses to NaCl and ABA stress). Notably, subcellular localization assays confirmed that CaBGLU21 resides in the tonoplast. Furthermore, qRT-PCR analysis revealed a characteristic transcriptional response pattern for *CaBGLU* genes under NaCl and ABA treatments, typically involving an initial decrease followed by an increase in expression. Collectively, these findings provide a crucial foundation for elucidating the functional roles and molecular mechanisms of the BGLU gene family in pepper growth, development, and stress adaptation.

## Data Availability

The data used for the analysis in this study are available in the article and the Supplementary Materials.

## Supplementary Materials

The following supporting information can be downloaded at: https://www.mdpi.com/article/10.3390/genes16080889/s1, Table S1: The primer sequences used in this study; Table S2: Information of BGLU gene family members in pepper.

## Abbreviations

The following abbreviations are used in this manuscript:

BGLU	β-Glucosidase
GH	glycoside hydrolase
